# Product News

**DOI:** 10.1155/S1463924681000618

**Published:** 1981


### Product News

**Editor's Note:-** *The address of the manufacturer/supplier appears in italics at the end of each item. In some cases this address will be that of a subsidiary to the manufacturing company as the address given is that from which the information has been obtained.*

### Emission spectroanalyser

Optical Emission Services has recently introduced a new emission spectroanalyser to the British market. The instrument is British made, is known as the OES 4500 and is available at a frequency of 27.12 mHz or as an arc-spark excitation source for solid samples. It permits the determination of up to 80 elements simultaneously and has a 1m rowland circle. The optical system is based on a holographic grating which is said to give good resolution and dispersion, wide spectral range, no ghosting and very low stray light characteristics.

The system is fully microprocessor automated and controlled and provides visual and printed copy of all data obtained.

*Optical Emission Services Ltd, 104 Tanners Drive, Blakelands, Milton Keynes.*

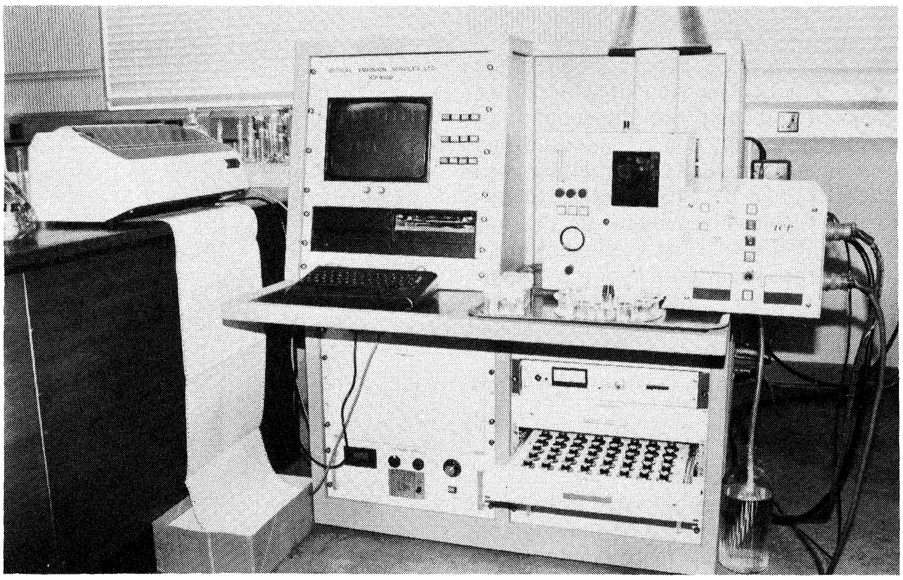

*Optical Emission Services' emission spectroanalyser*

### Nupro plug valves

Techmation is now supplying Nupro plug valves with handles in 316 stainless steel or aluminium. The handle is machined from ¾ inch hexagonal bar with a 316 roll pin inserted horizontally through the top.

When the roll pin is removed, the valve can be actuated with a socket wrench extension or an open-end wrench in high temperature or minimum clearance applications – and with the pin removed, there is protection against accidental opening and closing. The valve is quick acting, has a high capacity for straight through flow, a ¼ turn actuation and leak-tight shut-off. Operating temperature ranges from $-40$ to $204^\circ$C and the pressure rating is 2000 psi (20 600 kPa).

*Techmation Ltd, 58 Edgware Way, Edgware, Middx.*

### Flow injection analysis

In an agreement recently concluded with American Research Products, Plasma-Therm has been appointed as European agent for the ARPCO range of flow injection analysis equipment.

The AMFIA-2000 instrument is the result of development work carried out at the US Department of Agriculture and is described by Plasma Therm as the only completely automated system currently available. The operator programs the run using a simple interactive dialogue and thereafter, the instrument operates unattended until the next tray of samples has to be loaded. Analysis speeds of 150 - 360 samples per hour are possible, depending on the chemistry involved. Runs encompassing multiple sample trays (135 samples/tray) can be analysed from a single calibration curve. Microprocessor control allows both rapid operation and also easy transfer of methods from a manual or segmented regime onto the AMFIA-2000 and a ready facility for developing new methods. The two detector modules currently available include a filter photometer with a dynamic range of 0 - 3 absorbance units (spectral range 340 - 1200 nm) and a Fluro Tec digital filter photometer, wavelength range 254 – 650 nm which can also be applied in HPLC analysis.

Plasma-Therm is also to market the SMI Zeeman atomic absorption instrument in the UK. The instrument is made in West Germany by Erdmann and Grun and has been developed for detecting traces of toxic heavy metals such as Cd, Hg, Zn, As and Se. The Zeeman effect provides background compensation and allows measurements to be made even when smoke or other interferences are present.

*Plasma-Therm Ltd, 6 Station Rd, Penge, London SE20*

### Interface system

Ithaco has introduced a new model 488A interface system which provides the necessary interconnections for coupling a variety of Ithaco signal processing instruments to the IEEE 488A standard bus. The interface will simplify the use of Ithaco's pre-amplifiers and lock-in amplifiers in such applications as photoacoustic spectroscopy, photoelasticity studies, fibre optic characterisation studies and automatic thin film coating monitoring systems.

*Ithaco Inc, 735 West Clinton St, Ithaca, NY 14850, USA.*

### UV/vis spectrophotometer

There is a new UV/vis spectrophotometer available from Pye Unicam which the company says will be of particular interest to quality control laboratories. The SP7-500 double-beam scanning instrument has rapid keyboard operation, built-in alpha numeric printer, automatic audio-visual error warnings etc.

When used in colorimetric tests, the printer shows both the results and the settings with which they were obtained; in scanning mode the parameters are listed with the absorbance (or % T) and wavelength of the highest peak. A fault diagnosis system is incorporated.

*Pye Unicam Ltd, York St, Cambridge, CB1 2PX*

*SP7-500 UV/vis spectrophotometer from Pye Unicam*

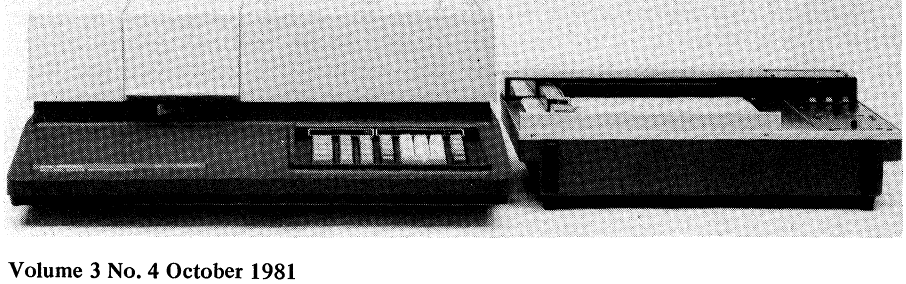

### Bench centrifuge

Baird & Tatlock has a new bench centrifuge on the market, the Mk IV Auto Bench. The centrifuge is British designed and built with design emphasis placed on safety, reliability, cost effectiveness and simplicity. It complies with ESCHLE, and BS 4402 (and is expected to meet the requirement of the new British Standard being produced) and is fitted with a lid interlock, out-of-balance cut-out and indicator, reinforced chamber, sealed buckets and a sealed chamber with outlet vent for ducting.

*Baird & Tatlock Ltd, PO Box 1, Romford Essex RM1 1HA*

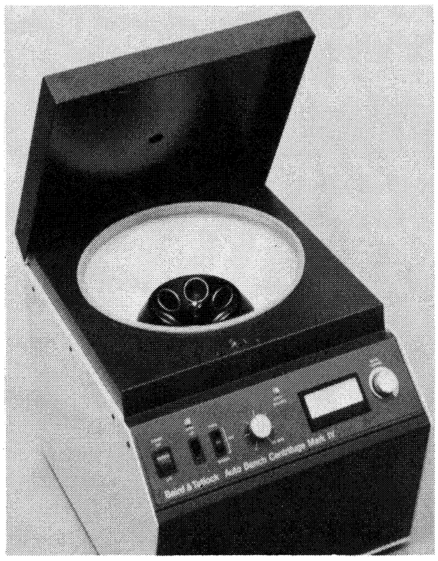

*Baird & Tatlock's Auto Bench mark IV*

### Gas chromatograph

Spectra-Physics has recently introduced a new generation of gas chromatograph, the SP 7100. The instrument can be used either as a stand-alone system or as part of a multiple GC network and has considerable potential for automation and central laboratory management.

A CRT/keypad interface is used for control and to show complete status of the instrument, including column flows.

The oven is large and has a rapid cool-down facility coupled to very low gradient specifications. Column installation and removal is said to be very easy. A combination of two packed and two capillary columns may be installed with side-by-side mounting of each type.

All types of capillary column can be used, and the capillary column head pressure - to 0.2 kPa – can be displayed on the CRT. Each detector has a specific contribution. The FID analyses 'dirty' samples – silyl derivaties and similar samples – without the need for frequent cleaning and calibration. The TCD has a linear response to 100% concentration in the cell.

The TCD for the detection of nitrogen and phosphorus is a completely new design. A dual channel data system, based on the established SP 4100 computing integrator, is available with the chromatograph which when linked with the optional alphanumeric keyboard gives a totally interactive system.

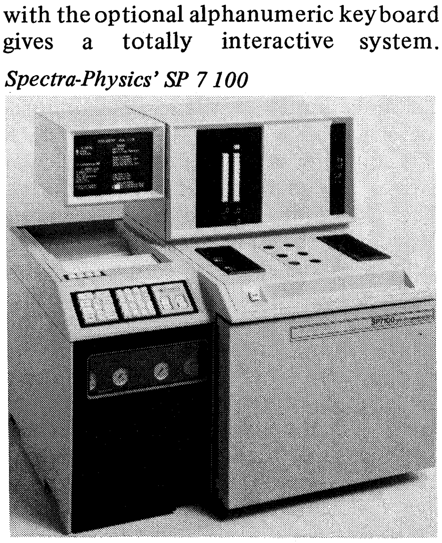

*Spectra-Physics' SP 7 100*

Another interesting feature is the ability to connect several instruments in a system network where each chromatograph can communicate and interact with all the others, or operate as a stand-alone instrument.

*Spectra-Physics Ltd, 17 Brick Knoll Park, St Albans, Herts*

### Computerised spectroscopy

Anaspec is supplying a micro-computer system for use in optical spectroscopy. The system is intended to handle data aquisition, signal averaging and spectrometer scan controls, including repeat scanning between two wavelength or frequency regions. Features include high level interactives software, graphic display of spectra, intensity data display, adjustable control 'BUG' and intensity and transmission or absorption readout facilities.

*Anaspec Ltd, PO Box 25, Newbury, Berks*

### Computerised emission spectrometry

New interface electronics and an extended choice of computer-controlled spectrometer configurations have been added to the Philips Range.

The PV 8665 interface is compatible with all Philips' spectrometers and source units, allowing flexible automation of measurement and system control. Of particular interest for ICP systems is the increased dynamic range of readout.

Modular construction enables systems to be tailored to users' needs with the option of later expansion. Simultaneous connection of up to 60 photomultipliers is possible. Photomultiplier signals are measured by integration, followed by transfer of intensity data to the computer for conversion into element concentrations. In the profiling mode, signals can be plotted on a chart recorder. The interface also allows software checking of various system voltages.

*Pye Unicam Ltd, York St, Cambridge CB1 2PX*

### HPLC sample preparation

Technicon Industrial Systems has developed an automated sample preparation system for use with HPLC, atomic absorption and other analytical techniques. The system automatically prepares, treats and collects solid or liquid samples. It offers filtration, dialysis, solvent extraction, evaporation-to-dryness, concentration, derivitisation and other capabilities.

*Technicon Instruments Corp, 511 Benedict Avenue, Tarrytown, New York 10591, USA*

### Programmable 2-phase lock-in amplifier

EG & G Princeton Applied Research has announced the microprocessor based Model 5206 two-phase lock-in analyser. Standard features include auto-phasing, auto-ranging and sine-wave response. An optional IEEE-488 interface card permits total programming and talk/listen operation with other devices on the bus. Full scale sensitivities range from 100 mV to 5 Vrms and operating frequencies span 0.2 Hz to 200 kHz. The amplifier should find application in measuring weak signal intensities, phase shifts and associated in-phase (X) and quadrature (Y) components. Results are presented in volts, dBs and degrees; also displayed are operating parameters such as internal oscillator frequency, DC output suppression, and phase-shifter settings. Two analogue meters augment the digital display to simultaneously allow trend following and the measurement of a variety of information all under the control of two pushbuttons. All processing, display modes and commands can be controlled by an external computer through the optional IEEE-488 card interface. Harmonic-free response at higher operating frequencies is now achieved without degraded dynamic-reserve (noise overload tolerance) and DC output stability specifications. Band selection is push-button and three band-determining cards can be simultaneously housed within the instrument to cover the operating range of 0.2 Hz – 200 kHz.

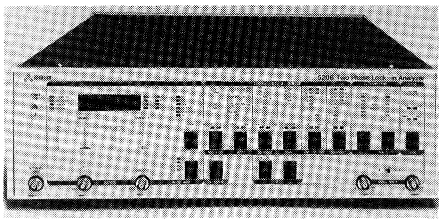

*EG & G Princeton Applied Research, PO Box 2565, Princeton, NJ 08540, USA*

### Flat field spectrometer

Schoffel/McPherson Instruments has designed a new flat field spectrometer, the model 251. The spectrometer uses toroidal gratings with an entrance focal distance of 292 mm. It features an ultra high vacuum capability, fixed width entrance slit, grazing incidence monochromator (at 70.6°) and it can be mounted in any physical orientation.

*Schoeffel/McPherson Instruments, 530 Main St, Acton, MA 01720, USA*

### Video spectrometer

ESI-Panax has combined a high performance single channel nuclear spectrometer with a Commodore PET to give a video spectrometer which with its powerful computing facility will allow the user to automate many routine measurements.

The spectrometer is the established 5000 series with all its timing and counting operations controlled by the computer. There is also a facility for scanning the analyser window automatically to give a multi-channel analyser type function. Timing intervals can be selected for single or repetitive counting, and the results displayed in numeric and graphic form on the CRT screen.

*UK agents: AID Scientific Ltd, Canford Works, Wallisdown Rd, Bournemouth, Dorset.*

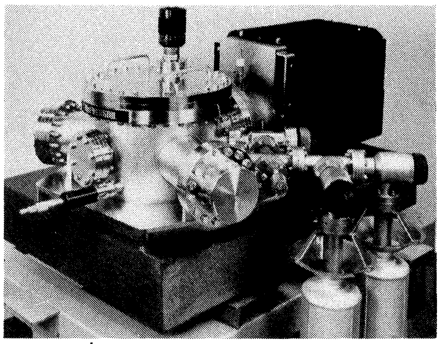

*Schoffel/McPherson's flat field spectrometer*

### Calcium analyser

Radiometer has introduced the ICA1 ionised calcium analyser, a computerised system capable of measuring ionised calcium and pH on 125 μl samples of whole blood, plasma or serum. In addition to direct measurements, the instrument calculates ionised calcium at pH 7.4. The measurements are performed by ion sensitive electrode with a calcium ion sensitive electrode, a pH electrode and a common reference electrode mounted in a measuring chamber thermostatted at 37°C. A cellophane membrane protects the sensitive part of the calcium electrode from protein contamination.

*Radiometer A/S, Emdrupvej 72, DK 2400, Copenhagen NV, Denmark.*

### Dual-beam LC detector

Perkin-Elmer's new LC-85 UV/vis spectrophotometer detector employs a double-beam optical system to give improved stability and compensation, said to be higher than the established LC-75.

The LC-85 allows stopflow scanning and other spectroscopic measurements at trace levels and a wide dynamic range improves quantitative linearity as well as facilitating use in prep-scale LC. An optional 2.4 μl flowcell accessory eliminates band broadening to give good efficiency in high speed LC.

*Perkin-Elmer Ltd, Post Office Lane, Beaconsfield, Bucks.*

### High power image analyser

Artek Systems has launched a computer based image analysis system which, it says, requires no special operator training. The model 800 image analyser has a 64 k byte memory linked to dual floppy disks and VDU screens. Operatoı instructions can be displayed on the left-hand screen at any time during an analysis while the right-hand screen is used to display the image fed to the analyser from a closed circuit television camera attached to the viewing microscope.

*Artek Systems Corporation, European Division, 32 Humberstone Rd, Cambridge, CB4 1JF*

